# LC-MS/MS Insight into Vitamin C Restoration to Metabolic Disorder Evoked by Amyloid β in *Caenorhabditis elegans* CL2006

**DOI:** 10.3390/metabo12090841

**Published:** 2022-09-06

**Authors:** Simeng Zhang, Yuchan Deng, Annan Zhang, Lili Yan, Zhichao Zhang, Jing Wei, Qiang Zhang

**Affiliations:** 1 Shaanxi Key Laboratory of Natural Products & Chemical Biology, College of Chemistry & Pharmacy, Northwest A&F University, Yangling 712100, China; 2 College of Biology Pharmacy & Food Engineering, Shangluo University, Shangluo 726000, China

**Keywords:** Alzheimer’s disease, dementia, neurodegenerative disease, aging, Aβ, metabolism, amino acid, ascorbic acid

## Abstract

The transitional expression and aggregation of amyloid β (Aβ) are the most important causative factors leading to the deterioration of Alzheimer’s disease (AD), a commonly occurring metabolic disease among older people. Antioxidant agents such as vitamin C (Vc) have shown potential effects against AD and aging. We applied an liquid chromatography coupled with tandem mass spectrometry (LC-MS/MS) method and differential metabolites strategy to explore the metabolic disorders and Vc restoration in a human Aβ transgenic (*Punc-54*::Aβ^1–42^) nematode model CL2006. We combined the LC-MS/MS investigation with the KEGG and HMDB databases and the CFM-ID machine-learning model to identify and qualify the metabolites with important physiological roles. The differential metabolites responding to Aβ activation and Vc treatment were filtered out and submitted to enrichment analysis. The enrichment showed that Aβ mainly caused abnormal biosynthesis and metabolism pathways of phenylalanine, tyrosine and tryptophan biosynthesis, as well as arginine and proline metabolism. Vc reversed the abnormally changed metabolites tryptophan, anthranilate, indole and indole-3-acetaldehyde. Vc restoration affected the tryptophan metabolism and the biosynthesis of phenylalanine, tyrosine and tryptophan. Our findings provide supporting evidence for understanding the metabolic abnormalities in neurodegenerative diseases and the repairing effect of drug interventions.

## 1. Introduction

Dementia is currently the seventh leading cause of death among all diseases, and causes disability and dependence among older people worldwide. The cognitive deterioration of dementia goes beyond the general consequences of biological aging. Dementia primarily affects older people, but it is not an inevitable consequence of aging. According to statistics and estimates from the World Health Organization, more than 55 million people live with dementia, with nearly 10 million new cases each year. The number is expected to rise to 78 million by 2030, and to 139 million by 2050. Moreover, the medical and care costs of dementia have a significant social and economic impact. In 2019, the total global social costs of dementia were estimated at USD 1.3 trillion; by 2030, these costs are predicted to exceed USD 2.8 trillion.

Alzheimer’s disease (AD) is the most common type of dementia, accounting for 60–70% of cases. An early symptom is short-term memory loss. AD patients can progress gradually to speech impairment, disorientation, emotional instability, loss of motivation, inability to take care of themselves and many behavioral problems [1]. In the brains of AD patients, amyloid β (Aβ) can aggregate to form amyloid plaques, while over-phosphorylated microtubule protein tau can aggregate to form neurofibrillary tangles. Aβ affects the phosphorylation state of tau by changing the balance between protein kinases and phosphatases [2]. Aβ can also promote the diffusion and absorption of tau in cells, thereby accelerating AD development [3]. Its hydrophobicity drives Aβ to self-aggregate easily and form oligomers. Aβ oligomers destroy synaptic function and lead to neuron death, ultimately affecting memory and cognitive functions. The appearance of Aβ oligomers and neuronal damage are speculated to occur decades before the arrival of clinical symptoms. Due to the critical role of Aβ in the development of AD, it is considered the most important biomarker of AD. Therefore, standard AD models are mainly constructed by introducing human Aβ genes in order to achieve the overexpression of Aβ and induce AD symptoms.

Among all AD animal models, transgenic *Caenorhabditis elegans* (worm) is the ideal animal model for exploring AD pathology and for use in the development of AD therapeutic drugs. The nervous system of nematodes contains the most molecular components of all higher animals, consisting of 302 neurons and 5000 chemical synapses [4]. According to bioinformatics analysis, nematode genes have 60–80% homology with human genes [5]. As an ideal model organism, *C. elegans* has many advantages, such as easy survival, high reproduction rate, short life span, complete transparency, short body and genome integrity. These characteristics make the worm a widely used and convenient model for neurobiology, developmental biology and anti-aging drug screening. Therefore, *C. elegans* can be an ideal model for exploring neurobiological molecular mechanisms [5,6]. The nematode CL2006 is a well-characterized and commonly used strain containing the *Punc-54*::Aβ^1–42^ transgene, which shows progressive, adult-onset paralysis [7].

The metabolome plays an essential role in neurodegenerative diseases. For example, the dysregulation of insulin causes a worsening of neurodegenerative diseases, including AD [8]. Leptin, another critical metabolic hormone, also plays a role in neurodegenerative diseases. A significant increase in cerebrospinal fluid (CSF) leptin levels was observed in AD patients compared to controls and patients with mild cognitive impairment, suggesting that leptin resistance develops throughout AD [9]. Metabolites are the endpoint of biological metabolism, and are thus directly related to the phenotype [10]. Since metabolites are altered when individuals are under pathological conditions, they can be used as biochemical and physiological indicators to help with early disease diagnosis [11]. Recently, the close relationship between metabolites and neurodegenerative diseases has been demonstrated in cells, animals and human bodies [12]. In addition, metabolic exploration also provides insight into drug effects with high-throughput profiling methods [13].

The presence of Vitamin C (Vc) can decrease Aβ oligomer formation [14]. AD pathological events involve ROS overproduction and extracellular Aβ42 deposition. Vc has been found to exert a range of protective effects against neurodegeneration in the brain caused by lipid peroxidation and endothelial dysfunction [15]. In this study, we applied the nematode strain CL2006 to explore metabolic variation associated with Aβ overexpression and metabolic restoration by Vc intervention. This will help to understand the metabolic changes in AD pathogenesis and Vc intervention, and will provide a basis for finding rational drugs from a metabolic perspective.

## 2. Materials and Methods

### 2.1. Chemicals

Vitamin C, reagents for the preparation of nematode growth media (NGM, including peptone, NaCl, CaCl2, MgSO4, cholesterol, KH2PO4 and K2HPO4), dimethyl sulfoxide (DMSO), Tween-20, Triton-100, Triisopropyl ethyl sulfonyl (Tris) and phosphate buffer were purchased from Solarbio Science & Technology Co., Ltd. (Solarbio, Beijing, China). Thioflavin S was purchased from Shanghai Yuanye Bio-technology Co., Ltd. (Yuanye, Shanghai, China). β-Mercaptoethanol was purchased from Shanghai Aladdin Bio-Chem Technology Co., Ltd. (Aladdin, Shanghai, China). Floxuridine was purchased from Shanghai Maclean Biochemical Technology Co., Ltd. (Maclean, Shanghai, China).

### 2.2. C. elegans Maintenance

The wild-type worm N2 (*C. elegans*) and transgenic strains CL2006 (dvIs2 [pCL12(unc-54/human Aβ peptide 1-42 minigene) + pRF4]) were purchased from the Caenorhabditis Genetics Center (University of Minnesota, MN, USA). The two strains were cultured and maintained on nematode growth media (NGM). N2 was propagated at 20 °C, while CL2006 was maintained at 15 °C [16]. Wild-type N2 and CL2006 were routinely propagated on solid NGM associated with the *Escherichia coli* OP50 strain that was used as the primary food source. Synchronous larvae were used for the experiments, which were acquired through bleaching egg-laying adults with a hypochlorite.

### 2.3. Paralysis Bioassay on C. elegans CL2006

The characteristic paralysis of CL2006 is caused by Aβ expression and accumulation in the cells in its body-wall muscle. When paralyzed, the worm can wave its head, but not its body. The paralysis status was evaluated according to a reported method [17]. To speed up the paralysis process, we cultured the worms at a higher temperature, 35 °C, for 1.5 h. After heat treatment, each worm group was transferred to NGM agar plates containing M9 buffer. Paralysis status was inspected by visual observation. Each worm was tapped with a bent platinum filament and evaluated for its ability to move. N2 wild-type nematodes did not exhibit significant paralysis. Student’s *t*-test was used for calculating the *p* values of CL2006 vs. Vc1, and for Vc1 vs. Vc2 contrasts.

### 2.4. Collection and Preparation of Metabolic Analysis Samples

We set up four parallel groups: a negative control (CL2006), the Vc-exposed groups (Vc1 and Vc2) and the wild-type N2 group as a blank control. Vc was dissolved in DMSO and diluted in *E. coli* OP50 liquid to a concentration of 50 µM and 100 µM while in use. The dilutions (70 µL) were then overlaid onto NGM plates (65 mm diameter) [18]. Each group had 9 replicates in parallel.

Age-synchronized transgenic CL2006 and N2 worms were fed on NGM solid plates, including different concentrations of Vc at 20 °C for 45 h. The worms were transferred to the new NGM solid plates containing Floxuridine (6 μM) and Vc, and incubated for 75 h. Approximately 2000 worms per replicate sample were gathered and washed 3 times with M9 buffer to remove OP50, then transferred to a 1.5 mL centrifuge tube to freeze rapidly with liquid N_2_. After being freeze-dried, each sample was extracted with 80% MeOH (200 μL per 1 mg dried worm) for 4 min in a JY92-IIDN cell disruptor (Ningbo Scientz Biotechnology Co., Ltd., Ningbo, China). The ultrasonic power of the disruptor was set to 20%. Each sample was then centrifuged at 12,000 rpm for 5 min at 4 °C. The supernatant was filtered through a syringe filter membrane (0.22 μm) before LC-MS/MS analysis.

### 2.5. UPLC-ESI-MS/MS Analysis

Chromatographic column: Accucore™ aQ C18 Polar Endcapped column, 150 × 2.1 mm, 2.6 µm (Thermos Fisher Scientific, Waltham, MA, USA); column temperature: 30 °C; flow rate: 0.3 mL/min; samples injection volume: 5.0 μL; mobile phase A: water with 0.1% (*v*/*v*) acetic acid; mobile phase B: acetonitrile with 0.1% (*v*/*v*) acetic acid. The column was eluted with gradient mobile phase B: it was kept at 2% B for 2 min, increased to 50% B after 5 min, then increased to 100% after 10 min and kept at 100% for 2 min. Before the next injection, the column was equilibrated at 2% B for 5 min.

The eluted metabolites were detected separately by positive and negative ion modes, with the Full MS-AIF method on a Q Extractive Focus instrument (Thermos Fisher Scientific, Waltham, MA, USA). Full MS resolution: 70,000; scan range: 70~1000 *m*/*z*; AGC target: 1.0 × 10^6^; spectrum data type: profile; AIF resolution: 70,000; AIF scan range: 70~1000 *m*/*z*; stepped NCE (normalized collision energy): 10 V, 20 V and 40 V; AIF spectrum data type: profile.

### 2.6. Metabolomics Data Analysis

The raw data files were analyzed following our previously reported procedure [19,20]. In brief, the positive and negative ion scan data files (*.raw) were imported into the software package MS-Dial version 4.90 [21]. Peaks were smoothed by the linear weighted moving average method, with smoothing level 3, minimum peak width 5; and minimum peak height 2 × 10^6^. For the metabolites identification process, we used the in-house reference MSP database, which integrated experimental HMDB (5.0) MSMS spectra [22] and CFM-ID predicted MSMS spectra [23,24] of KEGG compounds. The accurate mass tolerances were set to 2 mDa for MS1 and 5 mDa for MS2. All the sample data were aligned to QC data and normalized using the LOWESS method in MS-Dial. The processed result data were exported into one text file, which was then read by R script for further analysis and visualization. PCA and sPLD-DA were calculated using mixOmics [25]. The differential metabolites were filtered out under Fold Change (FC) > 2.0 and the *t*-test *p* < 0.02. KEGG pathway was enriched using the R package FELLA version 1.16.0 (Bioconductor project, Barcelona, Spain) [26].

## 3. Results

### 3.1. Inspection of Vc Effects against AD Paralysis

**Figure 1:**
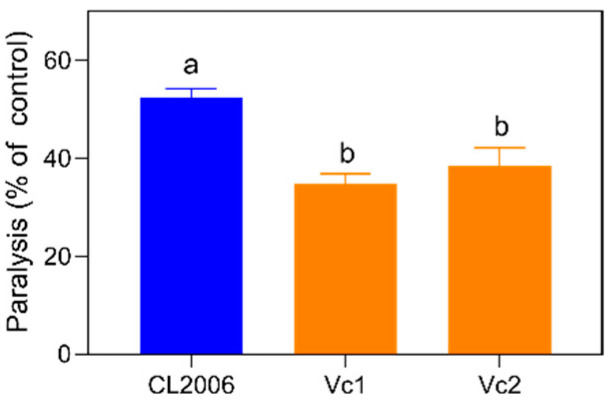
Paralysis rate in CL2006 exposed to Vc. Letters (a, b) in each column indicate statistically significant difference (*p* < 0.001).

The anti-AD effect of Vc has already been demonstrated, and Vc is generally used as a positive control in CL2006 bioassay [27]. We inspected the biological responses in CL2006 to Vc treatment in order to guarantee the validity of the subsequent metabolomic exploration. As shown in Figure 1, the heat treatment accelerated Aβ aggregation, and 52.4 ± 2.0% of worms exhibited significant paralytic symptoms. After being exposed to Vc, paralysis rates decreased significantly (*p* < 0.001) to 34.7 ± 2.1% (Vc1) and 38.4 ± 3.8% (Vc2). We found no statistical difference in paralysis rates between the two groups treated with Vc (Vc1 vs. Vc2).

### 3.2. Principal Component Analysis (PCA) of Metabolic Profiles

**Figure 2:**
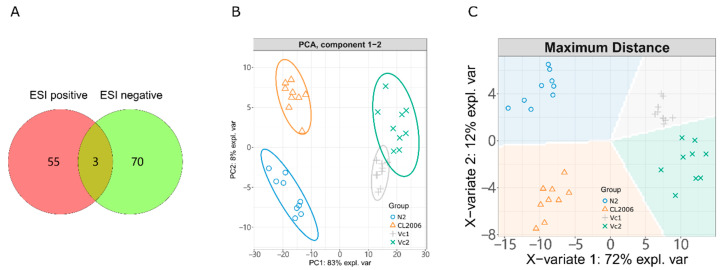
(**A**) the number of identified metabolites based on ESI positive and negative fragments. Detailed information on the identified metabolites can be found in Table S1 in the supplementary materials. (**B**) PCA and (**C**) sPLS-DA analysis.

We set up two blank groups (N2 wild-type and CL2006 strains), and two CL2006 groups exposed to Vc, that is, 50 µM Vc (Vc1) and 100 µM Vc (Vc2). Vc is often used as a positive control in CL2006 bioassays; it significantly inhibits Aβ protein aggregation and delays CL2006 paralysis [27]. We used UPLC-MS/MS to analyze metabolomic changes in CL2006 and Vc-treated CL2006. As shown in Figure 2A and Table S1, 128 physiologically important metabolites were detected from positive and negative ion scanning; these were identified by MS/MS fragment comparison with the HMDB MS database [22] associated with predicted MS, using the CFM-ID machine-learning model [23]. In the unsupervised principal component analysis (PCA, Figure 2B), all the normalized quantities of all identified metabolites were abstracted as two principal components (PCs), covering 91% of the features. PC1 represented 83% quantity features, and all the groups were mainly separated into three clusters: blank N2, blank CL2006 and Vc-exposed groups (Vc1 and Vc2). Vc exposure invoked a significant change in nematode metabolism. The two blank groups of N2 and CL2006 (Figure 2B) showed a pronounced difference, indicating different responses in the metabolome after Aβ was overexpressed. A supervised model of sparse partial least squares discriminant analysis (sPLS-DA) can afford more distinct features. As shown in Figure 2C, the sPLS-DA model gave distinctly separated predictions (background in Figure 2C) for those isolated groups of N2, CL2006, Vc1 and Vc2. The method also accounted for minor variations between approximate groups. Overall, meaningful differential metabolites could be mined from the distinct groups of N2, CL2006 and Vc1.

### 3.3. Metabolome Response to Aβ Overexpression and Aggregation

**Figure 3:**
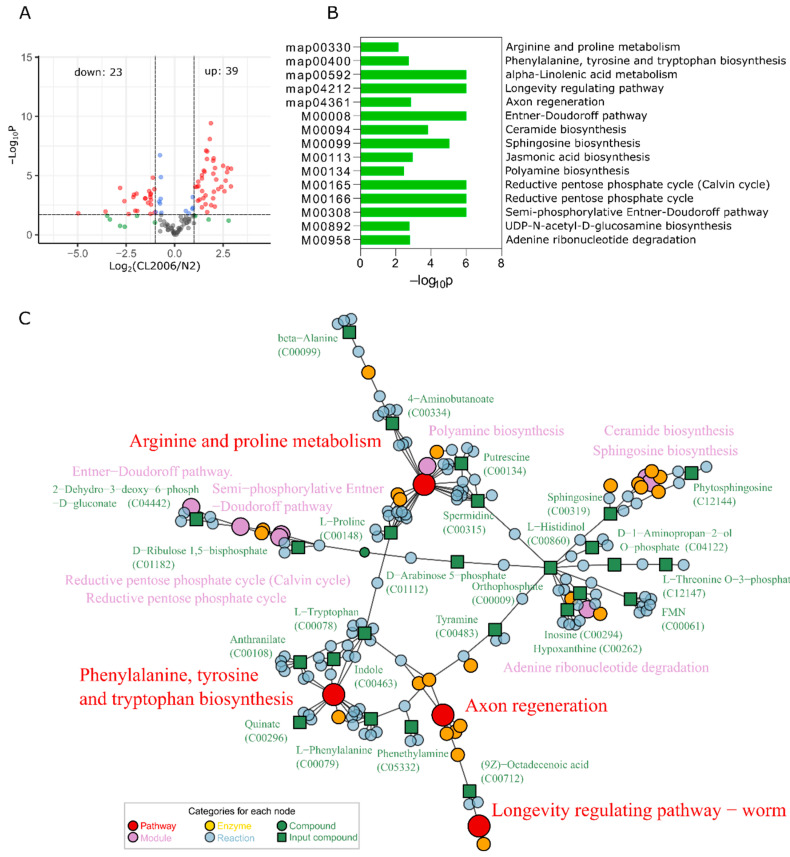
(**A**) Metabolite variation in the contrast CL2006 vs. N2 strains. Red scatter, FC > 2.0, *p* < 0.02; blue scatter, FC ≤ 2.0, *p* < 0.02; green scatter, FC > 2.0, *p* ≥ 0.02; grey scatter, FC ≤ 2.0, *p* ≥ 0.02. The relevant comparative data are shown in Table S2 in supplementary materials. (**B**) FELLA enrichment based on the differential metabolites. (**C**) Network relations of enriched KEGG nodes.

*Punc-54*::Aβ^1–42^ transgene stain (CL2006) invoked a significant metabolome response. Compared with the wild-type N2, the metabolome changed significantly after Aβ expression in CL2006, with 39 metabolites increasing and 23 metabolites decreasing (Figure 3A and Table S2). To annotate the functions of these metabolites, we performed an enrichment analysis using the Bioconductor package FELLA [26]. The enrichment was calculated based on KEGG pathways with the Diffusion method. All the enriched KEGG nodes with a *p* score of less than 0.02 are gathered in Figure 3B. Five signaling pathways and ten metabolic modules were obtained. The relevant pathways were map00330 (arginine and proline metabolism), map00400 (phenylalanine, tyrosine and tryptophan biosynthesis), map00592 (alpha-linolenic acid metabolism), map04212 (longevity-regulating pathway) and map04361 (axon regeneration).

The interrelationships among the enriched KEGG nodes (including pathways, modules, enzymes and reactions) are shown in Figure 3C. The two pathways, map00330 and map00400, had multiple matching metabolites. A total of five metabolites matched to the map00400 metabolic pathway. Their KEGG IDs were C00078 (L-tryptophan), C00079 (L-phenylalanine), C00108 (anthranilate), C00296 (quinate) and C00463 (indole). The biosynthesis and metabolism of phenylalanine and tryptophan involve the synthesis of neurotransmitters such as dopamine and serotonin. Four other metabolites matched to map00330: C00334 (γ-aminobutyric acid, GABA), C00134 (putrescine), C00148 (L-proline) and C00315 (spermidine). The pathway relationship network (Figure 3C) showed that the affected part in the map00330 was mainly on the synthesis module of polyamines (M00134).

### 3.4. Vc Repair of Aβ Metabolic Disorder

Vc has been reported to provide benefits in neurodegenerative disease, particularly AD [15]. This antioxidant was found to inhibit Aβ fibrillation resulting from AD development [28]. These reports prompted us to select Vc to treat CL2006, in order to explore potential restoration sites in metabolism.

**Figure 4:**
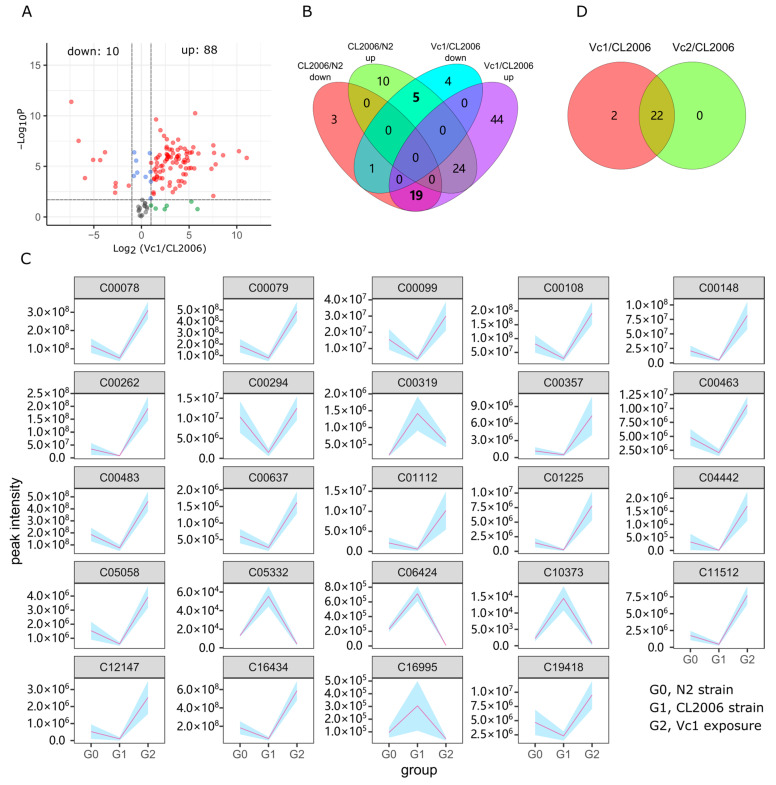
Vc restoration of metabolic disorder in AD. (**A**) Metabolite variation in the contrast of Vc1/CL2006. Red scatter, FC > 2.0, *p* < 0.02; blue scatter, FC ≤ 2.0, *p* < 0.02; green scatter, FC > 2.0, *p* ≥ 0.02; grey scatter, FC ≤ 2.0, *p* ≥ 0.02. The relevant comparative data are shown in Table S3 in supplementary materials. (**B**) The number distribution of up- and downregulated metabolites. (**C**) Trends of key metabolites, *p* < 0.02. Designators beginning with the letter C represent the KEGG compound ID. The shaded backgrounds under the lines represent the standard deviations. (**D**) The number of differential metabolites in the contrasts of Vc1/CL2006 and Vc2/CL2006, Fold Change > 2.0, *p* < 0.02.

After the culture temperature increased for the Vc-treated CL2006 groups (Vc1 and Vc2), the worm paralysis symptoms were significantly alleviated. In addition, the corresponding metabolome changed significantly compared to the CL2006 strain without Vc treatment, as shown in the PCA analysis (Figure 2). We filtered out changed metabolites with thresholds of FC > 2.0 and *p*-value < 0.02. As shown in Figure 4A and Table S3, Vc exposure invoked the upregulation of 10 and the downregulation of 88 metabolites. The Aβ-overexpressing CL2006 and the Vc treatment group Vc1 had both up- and downregulated metabolites. We summarize the number of these metabolites in the Venn diagram in Figure 4B. As shown in the Venn plot, five metabolites upregulated in CL2006 decreased after exposure to Vc culture (Vc1). Nineteen other metabolites which were downregulated in CL2006 showed the opposite tendency in the Vc treatment group. In other words, these 24 (5 + 19) differential metabolites were abnormally expressed when Aβ was overexpressed, then changed towards normal levels when the strain was treated with Vc, as shown in Figure 4C. We gathered basic information on these reversed metabolites, as shown in Table 1, including KEGG ID, formula, detected *m/z* and the normalized peak intensity for relative quantities. To further examine the reliability of these differential metabolites, we also exposed the CL2006 strain to a higher concentration (100 µM) of Vc in parallel (Vc2 group). Following the same data processing, we obtained 22 differential metabolites, as shown in Figure 4D. Exposure to a higher concentration of Vc did not result in a greater number of differential metabolites. Vc2 and Vc1 had most of the same differential metabolites. These differential metabolite changes are apparently due to Vc treatment.

**Figure 5:**
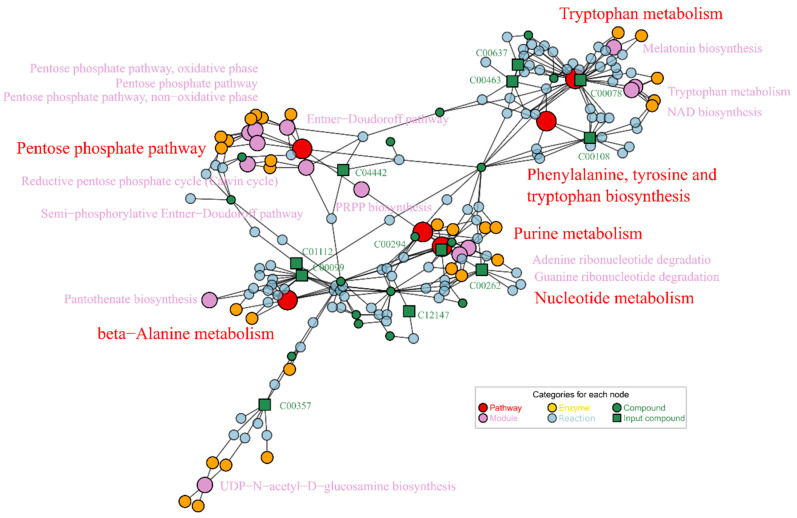
Enriched KEGG nodes based on differential metabolites of Vc1/CL2006 contrast.

We used these differential metabolites to perform enrichment, in order to annotate the functions of these metabolites related to Aβ and Vc treatment. The network displayed all the matched metabolites and enriched KEGG nodes (including pathways, modules and enzymes) in Figure 5. Not all metabolites could be enriched to extract relevant information in KEGG, since enrichment was limited by knowledge of the metabolite pathway and the KEGG database records. As shown in Figure 5, two of these pathways contain more matching metabolites: map00400 (phenylalanine, tyrosine and tryptophan biosynthesis) and map00380 (tryptophan metabolism). These two metabolic pathways (KEGG ID 00400 and 000380) are closely related, and their metabolic reactions and associated enzymes are intertwined. The differential metabolites that matched these two metabolic pathways were C00078 (L-tryptophan), C00108 (anthranilate), C00463 (indole) and C00637 (indole-3-acetaldehyde). The other two pathways, map00230 (purine metabolism) and map01232 (nucleotide metabolism), matched two metabolites: C00294 (inosine) and C00262 (hypoxanthine). All six matched metabolites showed noticeable changes among the three groups N2, CL2006 and Vc1, as shown in Figure 4C.

## 4. Discussion

### 4.1. Abnormal Aβ and Vc Exposure Led to Significant Variation in Metabolome in CL2006

In our LC-MS exploration, the metabolites identified from ESI positive- and negative-ions sources rarely overlapped (Figure 2A). Thus, the two ion modes (ESI+ and ESI–) can complement each other in metabolome analysis. Metabolome analysis can often reflect internal physiological changes in higher dimensions (metabolic features). Compared with wild-type N2 worms (Figure 2A,C), the Aβ abnormalities in CL2006 and in Vc treatment evoked significant changes in the overall metabolome. This metabolomic difference was consistent with the paralysis change in the phenotypic bioassay of CL2006 (Figure 1). However, different concentrations of Vc exposure (Vc1 and Vc2) did not lead to significant responses in paralysis or metabolomic features in PCA and sPLS-DA results (Figure 2B,C). Differences in metabolic characteristics were consistent with the results shown by the paralysis phenotype (Figure 1). Therefore, the characteristic metabolites associated with physiological changes can be mined from these different treatment groups.

### 4.2. Abnormal Aβ Affected Metabolism of Essential Substances Related to Nervous System

The differentially expressed metabolites among CL2006 and the wild N2 nematode were mainly involved in the synthesis and metabolism of amino acids, sphingolipids biosynthesis and the phosphorylation of pentose. In the Aβ-overexpressing strain CL2006, the synthesis of tryptophan (C00078) and phenylalanine (C00079) decreased, while GABA (C00334) increased. Their derivatives, serotonin and dopamine, were indicated as affected by abnormal Aβ.

In pathway enrichment analysis, matched metabolites are essential evidence for the affected pathway. The pathways of map00330 (arginine and proline metabolism) and map00400 (phenylalanine, tyrosine and tryptophan biosynthesis) had more matched metabolites than the other pathways (Figure 3C). Thus, the two pathways were indicated as the most influenced pathways. A number of different metabolites (13 in total) were matched onto the synthesis modules of ceramide, sphingosine and phosphate processes. Sphingosines are necessary for forming sphingolipids, which if disordered can also affect the function of neurons and is related to nervous system diseases [29]. In brief, Aβ overexpression interferes with the synthesis of neurotransmitters and destroys sphingolipid formation, thereby disturbing the primary action of neurons and leading to nerve dysfunction or neurodegenerative disease.

### 4.3. Vc Restored Mainly Phenylalanine, Tyrosine and Tryptophan Metabolism

Vc is a typical and widely recognized antioxidant. A growing number of cases demonstrate that Vc has a vital role in anti-aging and delaying the pathogenesis of Aβ [15]. In normal anti-AD drug screens, Vc is commonly used as a positive control agent in paralysis assays [27]. We therefore selected Vc as an anti-AD agent in order to explore its restorative effects on metabolome disorder and to obtain valuable information on metabolic regulation for AD treatment.

Aβ in CL2006 was activated during high-temperature culture, and the metabolites changed accordingly. Enrichment based on inversely changing metabolites showed that map00400 (phenylalanine, tyrosine and tryptophan biosynthesis) and map00380 (tryptophan metabolism) met more matched metabolites than the others. The two pathways were reported to be related to learning and memory impairment in mice treated with herbal medicines [30,31]. In our enrichment results, all four key metabolites (L-tryptophan, anthranilate, indole and indole-3-acetaldehyde) were in the pathway of tryptophan (map00380), while only anthranilate was simultaneously in the map00400 pathway. Both paths are related to tryptophan metabolism, and are therefore intertwined (Figure 5). This result suggests that the anti-AD effects of Vc treatment are primarily exerted through repair of the tryptophan metabolic pathway. Although genomic studies suggest that Aβ is associated with nucleotide variation [32] and purine metabolism [33], it is not clear which specific metabolites were specifically affected, nor has any of the literature determined whether anti-AD drugs reverse changes in these metabolites. This is the first revelation of a significant regulatory site where antioxidant reagents (represented by Vc) regulate metabolism in AD in vivo. Combined with phenotypic experiments, this metabolism regulation mitigates AD development and Vc treatment.

## 5. Conclusions

We investigated and mined the reverse changes of metabolites associated with Aβ activation and Vc restoration in CL2006. Aβ overexpression mainly changed the levels of metabolites in the biosynthesis and metabolism pathways of phenylalanine, tyrosine, tryptophan, arginine and proline. Vc treatment restored the 24 changed metabolites evoked by abnormal Aβ in CL2006. Vc treatment restored 24 of the Aβ-induced aberrant metabolites. In addition, enrichment revealed that tryptophan metabolism was the primary pathway affected by Vc. These findings provide a new metabolomic observation and interpretation of the pathogenesis of Aβ and the role of antioxidants in repairing metabolic disorders in AD.

## Figures and Tables

**Table 1:** Reversed metabolites among the groups N2, CL2006 and Vc treatment.

KEGG ID ^1^	Formula	Rt ^2^ (min)	Adduct	*m*/*z*	Peak Intensity (10^6^, Mean ± SD) ^3^		
					N2	CL2006	Vc Treated
C00078	C_11_H_12_N_2_O_2_	5.24	[M+H]^+^	205.0966	117.04 ± 39.79	49.13 ± 17.24	310.96 ± 47.16
C00079	C_9_H_11_NO_2_	2.45	[M+H]^+^	166.0858	184.9 ± 59.04	77.65 ± 27.81	486.3 ± 85.1
C00099	C_3_H_7_NO_2_	1.04	[M+H]^+^	90.0551	15.59 ± 6.36	3.64 ± 1.49	30.02 ± 8.87
C00108	C_7_H_7_NO_2_	5.63	[M+H−H_2_O]^+^	120.0443	80.85 ± 31.67	28.18 ± 9.91	192.18 ± 41.24
C00148	C_5_H_9_NO_2_	1.03	[M+H]^+^	116.0705	20.74 ± 9.09	4.74 ± 1.90	81.74 ± 23.89
C00262	C_5_H_4_N_4_O	1.39	[M+H]^+^	137.0455	34.54 ± 23.03	8.46 ± 1.95	191.87 ± 46.69
C00294	C_10_H_12_N_4_O_5_	1.89	[M−H]^−^	267.0731	10.31 ± 3.94	1.47 ± 0.94	12.49 ± 2.96
C00319	C_18_H_37_NO_2_	16.89	[M+H−H_2_O]^+^	282.2789	0.19 ± 0.03	1.42 ± 0.51	0.56 ± 0.14
C00357	C_8_H_16_NO_9_P	0.92	[M−H]^−^	300.0486	1.17 ± 0.67	0.49 ± 0.25	7.38 ± 3.39
C00463	C_8_H_7_N	5.24	[M+H]^+^	118.0650	4.82 ± 1.48	2.06 ± 0.64	10.65 ± 1.52
C00483	C_8_H_11_NO	2.45	[M+H−H_2_O]^+^	120.0806	186.1 ± 56.29	74.88 ± 25.63	462.15 ± 82.78
C00637	C_10_H_9_NO	5.24	[M+H−H_2_O]^+^	142.0647	0.60 ± 0.22	0.26 ± 0.10	1.62 ± 0.33
C01112	C_5_H_11_O_8_P	0.91	[M−H]^−^	229.0115	2.09 ± 1.37	0.55 ± 0.46	10.20 ± 4.76
C01225	C_9_H_19_O_11_P	0.90	[M−H]^−^	333.0595	1.45 ± 0.78	0.24 ± 0.20	7.80 ± 2.48
C04442	C_6_H_11_O_9_P	0.91	[M−H]^−^	257.0061	0.33 ± 0.31	0.01 ± 0.03	1.70 ± 0.56
C05058	C_6_H_7_NO	5.63	[M+H−H_2_O]^+^	92.0496	1.54 ± 0.64	0.6 ± 0.13	3.93 ± 0.77
C05332	C_8_H_11_N	5.39	[M+H]^+^	122.0964	0.01 ± 0.00	0.06 ± 0.01	0.00 ± 0.00
C06424	C_14_H_28_O_2_	16.22	[M−H]^−^	227.2006	0.23 ± 0.04	0.71 ± 0.10	0.01 ± 0.00
C10373	C_18_H_28_O_4_	11.63	[M−H]^−^	307.1909	0.00 ± 0.00	0.01 ± 0.00	0.00 ± 0.00
C11512	C_13_H_20_O_3_	10.59	[M−H]^−^	223.1332	1.75 ± 0.69	0.45 ± 0.22	7.72 ± 1.36
C12147	C_4_H_10_NO_6_P	0.91	[M+AcO]^−^	258.0381	0.52 ± 0.43	0.10 ± 0.10	2.53 ± 0.97
C16434	C_6_H_13_NO_2_	1.47	[M+H]^+^	132.1015	183.22 ± 70.92	64.32 ± 20.31	587.5 ± 105.3
C16995	C_17_H_34_O_2_	17.53	[M−H]^−^	269.2479	0.10 ± 0.04	0.30 ± 0.20	0.04 ± 0.01
C19418	C_18_H_34_O_3_	12.31	[M−H]^−^	297.2432	4.69 ± 2.24	2.31 ± 0.81	9.52 ± 2.45

^1^ The name, chemical structure and other basic information of metabolites can be acquired from https://www.kegg.jp/kegg/compound/ (accessed on 16 July 2022). ^2^ Rt, retention time (min) in chromatography. ^3^ Peak intensities (in 10^6^ unit) were normalized by LOWESS method in MS-Dial (Riken center for sustainable resource science, Kanagawa, Japan).

## Data Availability

The data presented in this study are available in article and supplementary materials.

## Supplementary Materials

The following supporting information can be downloaded at: https://www.mdpi.com/article/10.3390/metabo12090841/s1, Table S1: All identified metabolites; Table S2, Differential metabolites (CL2006 vs. N2); Table S3, Differential metabolites (Vc1 vs. CL2006). Metabolite names with * were identified by predicted spectra of CFM-ID.
